# Silymarin enhances the response to oxytetracycline treatment in *Oreochromis niloticus* experimentally infected with *Aeromonas hydrophila*

**DOI:** 10.1038/s41598-023-43270-z

**Published:** 2023-09-27

**Authors:** Ahmed H. Sherif, Adel E. Toulan, Naglaa El-kalamwi, Enas A. H. Farag, Abeer E. Mahmoud

**Affiliations:** 1 Fish Diseases Department, Animal Health Research Institute AHRI, Agriculture Research Center ARC, Kafrelsheikh, 12619 Egypt; 2 Sakha Unit, Central Laboratory for Aquaculture Research, Agriculture Research Center ARC, Kafrelsheikh, Egypt; 3 Pathology Department, Animal Health Research Institute AHRI, Agriculture Research Center ARC, Dokki, 12619 Egypt; 4 Department of Pharmacology, Animal Health Research Institute AHRI, Agriculture Research Center ARC, Benha, 12619 Egypt; 5 Fish Diseases Department, Animal Health Research Institute AHRI, Agriculture Research Center ARC, Dokki, 12619 Egypt

**Keywords:** Immunology, Microbiology, Zoology

## Abstract

Many governments have approved the use of oxytetracycline as an antibiotic additive to food fish, with oxytetracycline now routinely used in many nations. However, oxytetracycline is known to have immunosuppression impacts. We, therefore, evaluated the immunological, antioxidative, and histopathological status of Nile tilapia fed a diet containing silymarin (100 mg/kg fish feed) for 0, 2, 4, 6, and 8 weeks. The protective effects of silymarin against *Aeromonas hydrophila* (*A. hydrophila*) infection and oxytetracycline treatment were evaluated. Blood parameters (erythrocyte count, white blood cell count, hemoglobin, and packed cell volume) improved over time in fish fed on dietary silymarin. Serum levels of alanine aminotransferase (ALT) were lower in fish fed on dietary silymarin, whereas serum levels of aspartate transferase (AST)and alkaline phosphatase (ALK) were unchanged. Dietary silymarin affected serum lipid profiles as decreases in serum triglyceride and low-density lipoprotein cholesterol levels and a trend toward lower cholesterol levels, whereas serum high-density lipoprotein cholesterol levels were increased compared to fish fed on the control diet. Dietary silymarin resulted in an increase of serum total protein levels and globulin fractions. Significant and progressive increases in catalase and glutathione peroxidase levels were observed after six weeks of feeding on a dietary silymarin before decreasing to control levels at the end of the experimental period. Fish fed on dietary silymarin, interleukin-1 and fish tumor necrosis factor-alpha were upregulated in hepatic tissues; however, interleukin-10 levels decreased to comparable levels to controls after eight weeks. Fish infected with *A. hydrophila* displayed septicemia (opaque eye, hemorrhagic ulcers, dentated fins, hepatomegaly, and splenomegaly). Reduced mortality was observed in Nile tilapia infected with *A. hydrophila* and fed a diet containing silymarin, indicating that silymarin improves fish responses to oxytetracycline with a 37% reduction in mortality.

## Introduction

Silymarin is a flavonolignan derived from the seed of milk thistle (*Silybum marianum* L*.*) that contains silybinin A and B, isosilybinin A and B, and silydianin^1,2^. Given its phytotherapeutic characteristics, silymarin has been widely employed as a fish feed additive for the treatment and prevention of diseases in recent years^3,4^. Dietary silymarin reportedly improves the growth performance of grass carp *(Ctenopharyngodon idellus)*^5^ and increases the activity of superoxide dismutase (SOD), an antioxidant enzyme, in the liver of turbot *(Scophthalmus maximus* L.)^6^.

The liver is the primary organ responsible for both endogenous and external chemical metabolism and one of the first organs to be affected by toxic exposures^7^. Silymarin, a hepatoprotective antioxidant with anti-lipid and anti-inflammatory properties, has been shown to have hepatoprotective effects in common carp *(Cyprinus carpio)*^8^. The steroid structure of silymarin may alter the hepatocyte cell membrane by blocking the entry of xenobiotics and trapping free radicals, thereby increasing intracellular concentrations of glutathione and inhibiting lipid peroxidation^9,10^. Previous studies have examined the effects of silymarin on juvenile grass carp at doses of 0, 100, and 200 mg/kg^5^. Although compounds derived from plants may be used for chemotherapy, compounds used as supplements in fish diets must undergo physiological and biological examinations before their routine use.

The rapid rise of the global aquaculture sector over the last seven decades has generated a multibillion-dollar business (aquatic animal protein and global food security)^11^. The intensification of aquaculture to meet the growing demand for seafood has led to an increase in the prevalence of infectious diseases among fish stocks, with increasing stock densities leading to increased transmission of pathogens. Further, decreased water quality has been shown to induce stress factors that impact disease persistence within fish. The annual cost of infectious disease epidemics among fish stocks has been reported as 6 billion USD^12^.

Only three antibiotics used in fish are Food and Drug Administration approved and commercially available in the United States: oxytetracycline (OTC, Terramycin for Fish, Phibro Animal Health, Inc., Fairfield, NJ); florfenicol (Aquaflor, Intervet/Schering-Plough, Animal Health Corp. Summit, NJ); and sulfadimethoxine and ormetoprim (Romet-30, Pharmaq AS, Oslo, Norway)^13^. Tetracyclines have broad-spectrum bacteriostatic action which prevents the growth of both Gram-negative and Gram-positive bacteria by inhibiting bacterial protein synthesis. Tetracyclines have been widely employed in aquaculture due to excellent penetration into bodily fluids and tissues, and low cost^14,15^. As tetracyclines are inexpensive, simple to administer, and cause less stress to fish, the oral route is frequently used for the administration of tetracyclines to aquatic animals, with in-feed oxytetracycline widely used in the treatment of Nile tilapia (*Oreochromis spp*)^16–18^. Many studies have reported that oxytetracycline has histopathological, immunosuppressive, and genotoxic characteristics in farmed fish species, including rainbow trout^19,20^. Accordingly, dietary supplementation is required to limit oxytetracycline withdrawal and increase fish responses to oxytetracycline treatment.

## Materials and methods

### Experimental design

In the present study, two hundred Nile tilapia (*O. niloticus*) with an average weight of 50 ± 3 g were collected from local freshwater fish farms (Tolompat7 village in Kafrelshiekh governorate Egypt) and transferred to the wet laboratory at Animal Health Research Institute at Kafrelsheikh. Fish were maintained in a water tank for acclimation for two weeks. Fish were distributed into fifteen glass aquaria (aquarium size: 50 × 50 × 40 cm) after sedation with tricaine methanesulfonate. Antiseptic therapy was administered befor transfer into new aquaria^21,22^. Silymarin was used as a feed additive at a dose of 0.1% (1 g per kg of dry ration)^23^ for 2, 4, 6, and 8 weeks, and were abbreviated Sil-0, Sil-2, Sil-4, Sil-6, and Sil-8, respectively. Fish were divided into two groups. The control group was maintained in ten aquaria (10 fish per aquarium). The second group (100 fish) was maintained in ten glass aquaria. Blood and tissues samples were collected at 0, 2, 4, 6, and 8 weeks. Fish were challenged with *Aeromonas hydrophila* (*A. hydrophila*) via intraperitoneal injection at a dose of 2.4 × 10^5^ CFU/mL at 0, 2, 4, 6, and 8 weeks and then treated with dietary oxytetracycline (83 mg/kg b w) for 10 days^24^.

Commercial fish food (in the form of pellets) was soaked in water and blended into a paste, to enhance the consistency and viscoelastic properties of the food, gelatin (Nutri-B-Gel) produced by Canal Aqua Cure (Port-Said, Egypt) was mixed with the formed paste to a final level of 5% w/w. The diet pastes were sited into syringes that yielded a 2 mm-thick diet. The extruded diets were left to dry before cutting into similar size pellets. Fish feed was provided twice a day at 08.00 am and 02.00 pm. The amount of feed was changed weekly to provide a weight of feed corresponding to 3% body weight^25^.

Silymarin (capsules, 140 mg) was purchased from a local market. Silymarin capsules were manufactured by Safe for pharmaceutical products, new Borg El-Arab, Egypt, packed by South Egypt Drug Industries Company SEDICO (Batch No.: 0920468/2020). Oxytetracycline (powder, 20%) was purchased from a local market produced by the Arab Company for Gelatin and Pharmaceutical Products (Arab Caps) under registration no. (M.O.H. Reg. No.: 838/2004).

### Serological analyses of experimental fish

Blood samples were collected after the anaesthetization of fish with tricaine methanesulfonate (MS222; Sigma, St. Louis, MO, USA). Blood samples were drawn from the caudal vein of experimental Nile tilapia using a syringe coated with heparin (100 IU/ml) and without anticoagulant to obtain whole blood and serum, respectively. Red blood cell (RBC) and white blood cell (WBC) counts were obtained using a hemocytometer^26^. Packed cell volume (PCV) was determined using the microhematocrit technique. Serum hemoglobin (Hb) levels were calculated using the cyanmethemoglobin method^27^. All serum samples were stored at -80 °C till analyses were performed.

### Liver enzymes and lipid profile in fish serum

Hepatic enzymes aspartate aminotransferase (AST), alanine aminotransferase (ALT), and alkaline phosphatase (ALK) levels were measured colorimetrically^28^ using kit reagents supplied by Diamond Diagnostic Co. (Holliston, USA). Lipid profiles were colorimetrically measured in experimental fish serum including triglycerides (TG)^29^, cholesterol (CH)^30^, high-density lipoprotein cholesterol (HDL)^31^, and low-density lipoprotein cholesterol (LDL)^32^.

### Serum protein levels in Nile tilapia

Serum concentrations of total protein (TP)^33^ and albumin (ALB)^34^ were measured by colorimetric methods. Globulin (GLO) concentrations were calculated by subtracting the ALB concentration from the concentration of TP. The electrophoretic pattern of serum α, β, and γ GLOs was assessed using polyacrylamide gel columns^35^. Gels were imaged and analyzed according to previously described methods^36^.

### Hepatic antioxidant enzyme levels in Nile tilapia

The levels of glutathione peroxidase (GPx, EC 1.11.1.9) and catalase (CAT; EC 1.11.1.6) in hepatic tissue of experimental Nile tilapia were determined^37,38^.

### Serum cytokine levels in experimental fish

Serum levels of interleukin-1β (IL-1β), interleukin-10 (IL-10), and fish tumor necrosis factor-alpha (TNF-α) were measured using solid-phase sandwich enzyme-linked immunosorbent assay (ELISA) test kits (My BioSource Co., San Diego, California, USA). ELISA was performed according to the manufacturer’s protocols.

### Infective signs, mortality rate, and relative level of protection

Experimental fish were challenged with virulent *A. hydrophila* (AHRAS2) at 0, 2, 4, 6, and 8 weeks. At each time point, fish were divided into two groups, one group was treated with oxytetracycline for ten days while the other group received no antimicrobial treatment. Each fish was intraperitoneally administered 0.2 mL of a bacterial suspension containing 2.4 × 10^5^ CFU/mL in phosphate buffer saline using the McFarland scale. *A. hydrophila* (AHRAS2)^39^ and deposited in GenBank with the accession number MW092007, *A. hydrophila* expresses cytotoxic enterotoxin genes (*act* and *alt*). Mortality rate (MR) and relative level of protection (RLP) were determined in all groups. MR (%) was estimated according to the following formula.

$${\text{MR }}\left( \% \right) = \left( {{\text{No}}.{\text{ of deaths in a specific period}} \div {\text{total population during that period}}} \right) \times 100$$

Meanwhile, RLP was calculated in challenged fish^40^ as follows:

$${\text{RLP }}\left( \% \right) = \left( {{1}{-}\left( {\% {\text{ mortality in the treated group}} \div \% {\text{ mortality in the control group}}} \right)} \right) \times 100$$

### Histopathology

From experimental fish, liver samples were collected and fixed in 10% buffered formalin to assess the impact of oxytetracycline and the ameliorative role of silymarin. After alcohol dehydration and xylol clearing, specimens were embedded in paraffin, then cut into sections and stained with haematoxylin–eosin for microscopic examination^41^.

### Statistical analyses

The impact of silymarin on the health of Nile tilapia was evaluated by determining the mean and standard error of the collected data. Groups were compared using ANOVA and Duncan's Multiple Range^42^ using SPSS software^43^. *P* values less than 0.05 were considered statistically significant.

### Biosafety

Dead fish were burned in fixed incinerators at the laboratory. The present study followed the biosafety measures concerning pathogen safety described by the Pathogen Regulation Directorate (Infectious substances—*A. hydrophila* datasheet)^44^.

### Ethical approval

The above described methodology was approved by the Ethics Committee at the Animal Health Research Institute and European Union directive 2010/63UE, and all methods were carried out in accordance with relevant guidelines and regulations. This study is reported in accordance with ARRIVE guidelines (https://arriveguidelines.org). This paper does not contain any studies with human participants by any of the authors. No specific permissions were required for access to the artificial pond in wet laboratory Animal Health Research Institute, Kafrelsheikh, Egypt. The field studies did not involve endangered or protected species.

## Results

### Serological parameters

Nile tilapia fed a dietary silymarin had improvements in serological indices at four weeks (RBC, 6.45 × 10^6^; WBC, 49.27 × 10^3^; Hb, 14.13 g/dl; PCV, 45.57%; Table 1) compared to the control. No significant differences were observed between time points or groups. The survival rate (SR; %) of experimental Nile tilapia was higher in silymarin-fed fish at two weeks (95%) compared to baseline (90%) and remained higher than baseline until the end of the 8-week experimental period.

**Table 1:** Serological parameters and survival rate.

Items	RBC (× 10^6^)	WBCs (× 10^3^)	Hb (g/dl)	PCV (%)	SR (%)
Sil-0	2.31 ± 0.1^C^	35.7 ± 2.45^C^	9.46 ± 0.4^C^	30.49 ± 1.28^C^	27/30 (90)
Sil-2	3.13 ± 0.12^AB^	42.67 ± 1.23^B^	12.85 ± 0.5^AB^	41.37 ± 1.59^AB^	95/100 (95)
Sil-4	3.45 ± 0.18^A^	49.27 ± 2.66^A^	14.13 ± 0.74^A^	45.57 ± 2.38^A^	80/85 (94.11)
Sil-6	2.87 ± 0.12^B^	38.3 ± 1.96^BC^	11.75 ± 0.5^B^	37.8 ± 1.6^B^	67/70 (95.71)
Sil-8	2.75 ± 0.08^B^	41.1 ± 0.67^BC^	11.28 ± 0.31^B^	36.35 ± 0.99^B^	55/57 (96.5)

Superscript letter within the same column indicate significant differences between values at *P* ≤ 0.05. Sil-0, control group; Sil-2, group fed silymarin-containing diet for 2 weeks; Sil-4, 4 weeks; Sil-6, 6 weeks; Sil-8, 8 weeks; RBC, red blood cell; WBC, white blood cell; Hb, hemoglobin; PCV, packed cell volume.

### Serum enzymes and lipid profiles

**Figure 1:**
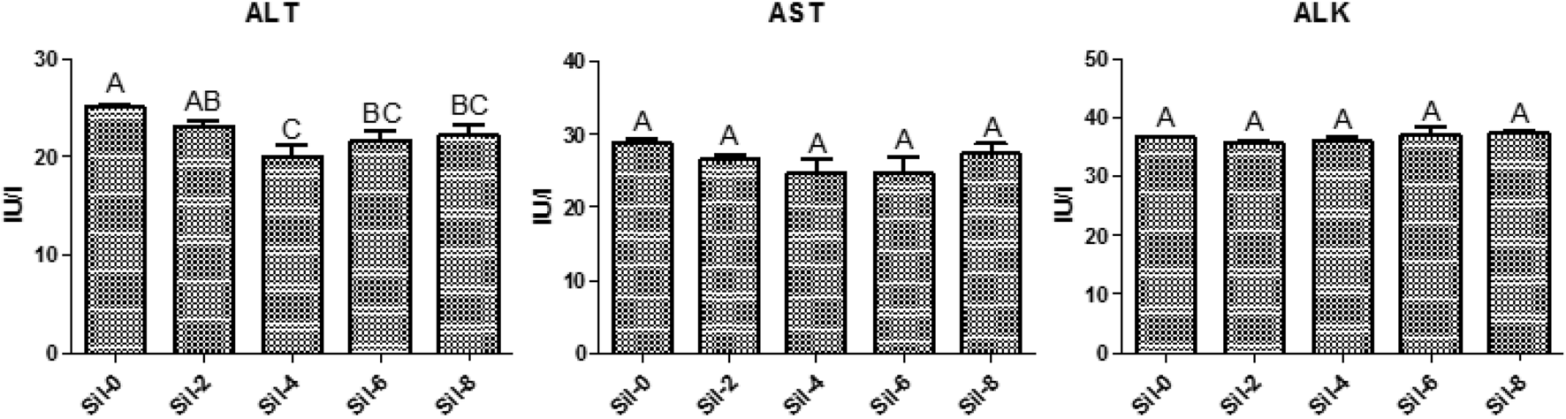
Serum liver enzyme levels and lipid profiles. Different letters indicate significant differences between values at *P* ≤ 0.05. Sil-0, control group; Sil-2, group fed silymarin-containing diet for 2 weeks; Sil-4, 4 weeks; Sil-6, 6 weeks; Sil-8, 8 weeks; ALT, alanine aminotransferase; AST, Aspartate aminotransferase; ALK, alkaline phosphatase.

Serum ALT levels were significantly decreased in Nile tilapia fed on a silymarin-containing diet for four weeks compared to baseline (4 weeks, 20 IU/l vs. baseline, 25.07 IU/l). No significant differences in serum ALT levels were observed between other time points. No significant differences in serum levels of AST or ALK were observed between time points (Fig. 1).

**Figure 2:**
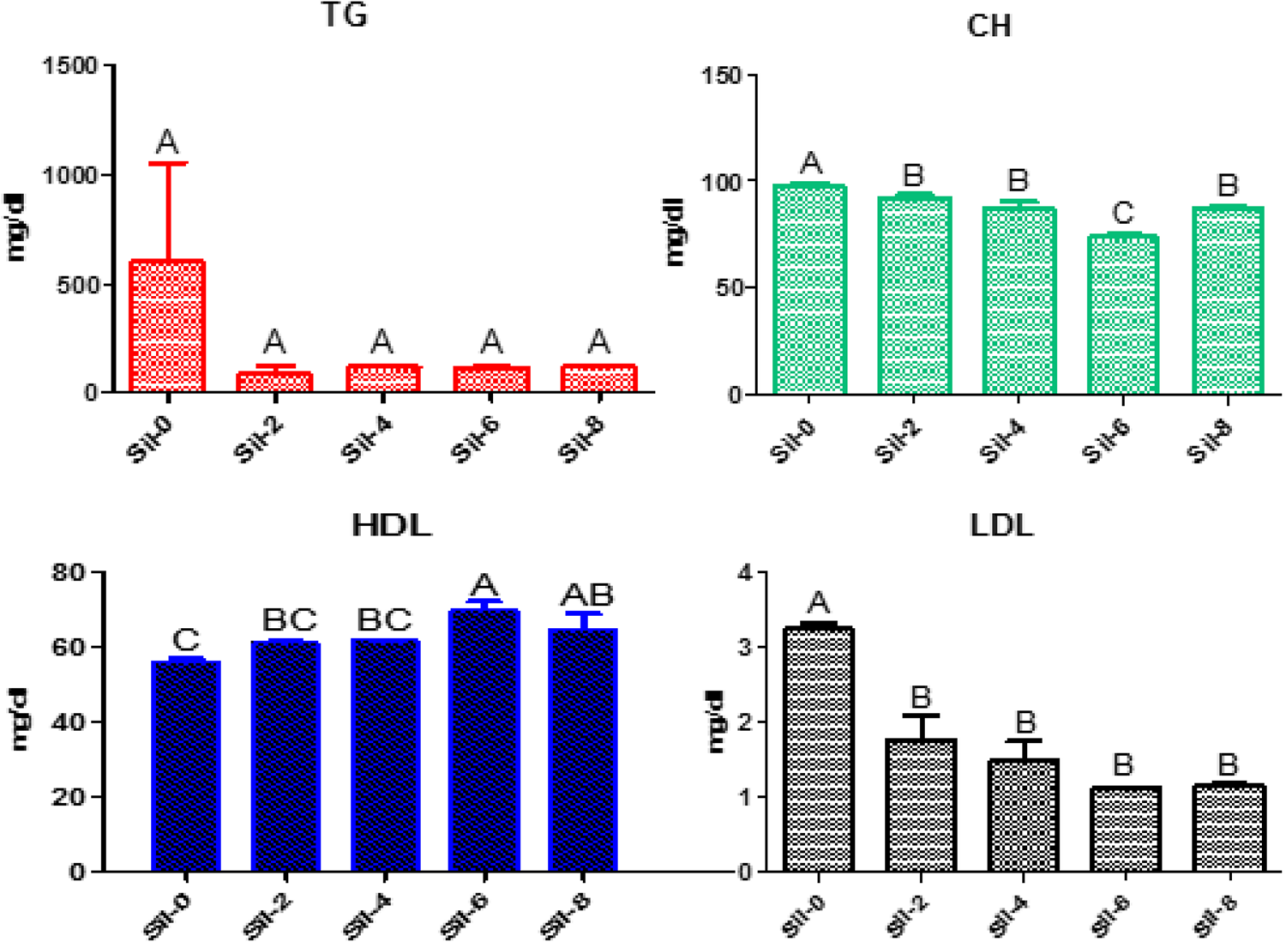
Serum lipid profiles. Different letters indicate significant differences between values at *P* ≤ 0.05. Sil-0, control group; Sil-2, group fed silymarin-containing diet for 2 weeks; Sil-4, 4 weeks; Sil-6, 6 weeks; Sil-8, 8 weeks; TG, triglycerides; CH, cholesterol; HDL, high-density lipoprotein (HDL) cholesterol; and LDL, low-density lipoprotein cholesterol.

No significant differences in serum lipid profiles in Nile tilapia including TG, CH, HDL, and LDL levels were observed between timepoints. CH levels were significantly lower than baseline after tilapias were fed a silymarin-containing diet for 6 weeks. Serum HDL levels were significantly increased at six weeks compared to baseline (6 weeks, 70.17 mg/dl vs. baseline, 56.4 mg/dl; Fig. 2).

### Serum protein levels and globulin electrophoresis

Serum TP levels were significantly increased from baseline to 5.74 g/dl in Nile tilapia fed a silymarin-containing diet for 6 weeks. Significant increases in serum ALB levels at four weeks (2.95 g/dl); however, no significant differences in serum ALB levels were observed between 8 weeks (2.73 g/dl) and baseline (2.76 g/dl).

Serum GLO levels were increased in fish fed a silymarin-containing diet compared to baseline values. Alpha GLO levels (1.03 g/dl) and beta GLO levels (0.81 g/dl) were significantly increased at 6 weeks compared to baseline (0.64 g/d and 0.46 g/dl, respectively). Gamma GLO levels were significantly increased at four weeks compared to baseline (4 weeks, 0.4 g/dl vs. baseline, 0.62 g/dl; Table 2).

**Table 2:** Serum protein analyses.

Items	TP (g/dl)	ALB (g/dl)	GLO (g/dl)	GLO-α (g/dl)	GLO-β (g/dl)	GLO- γ (g/dl)
Sil-0	4.56 ± 0.05^D^	2.76 ± 0.03^B^	1.8 ± 0.02^C^	0.64 ± 0.03^D^	0.46 ± 0.03^C^	0.4 ± 0.02^C^
Sil-2	5.17 ± 0.02^C^	2.71 ± 0.02^B^	2.46 ± 0.01^B^	0.83 ± 0.01^C^	0.71 ± 0.01^B^	0.6 ± 0.02^AB^
Sil-4	5.47 ± 0.05^B^	2.95 ± 0.03^A^	2.52 ± 0.04^B^	0.88 ± 0.003^BC^	0.72 ± 0.01^B^	0.62 ± 0.03^A^
Sil-6	5.74 ± 0.05^A^	2.97 ± 0.08^A^	2.76 ± 0.04^A^	1.03 ± 0.04^A^	0.81 ± 0.01^A^	0.63 ± 0.07^A^
Sil-8	5.21 ± 0.05^C^	2.73 ± 0.06^B^	2.47 ± 0.02^B^	0.94 ± 0.04^B^	0.74 ± 0.02^B^	0.49 ± 0.01^BC^

Superscript letters within the same column indicate significant differences between values at *P* ≤ 0.05. Sil-0, control group; Sil-2, group fed silymarin-containing diet for 2 weeks; Sil-4, 4 weeks; Sil-6, 6 weeks; Sil-8, 8 weeks; TP, total protein; ALB, albumin; GLO, globulin.

### Antioxidant responses

In Nile tilapia fed a silymarin-containing diet, hepatic levels of GPx and CAT reached a maximum level at four weeks (GPx, 5.3 U/mg; CAT, 2.99 U/mg) compared to baseline (GPx, 2.29 U/mg; CAT, 0.61 U/mg) and then decreased at eight weeks (GPx, 2.33 U/mg; CAT, 1.12 U/mg), as shown in Table 3.

**Table 3:** Glutathione and catalase levels in hepatic tissues.

Items	GPx (U/mg prot)	CAT (U/mg prot)
Sil-0	2.29 ± 0.1^C^	0.61 ± 0.07^D^
Sil-2	3.41 ± 0.08^B^	1.32 ± 0.07^C^
Sil-4	5.3 ± 0.58^A^	2.99 ± 0.06^A^
Sil-6	3.09 ± 0.06^BC^	1.84 ± 0.06^B^
Sil-8	2.33 ± 0.18^C^	1.12 ± 0.13^C^

Superscript letters within the same column indicate significant differences between values at *P* ≤ 0.05. Sil-0, control group; Sil-2, group fed silymarin-containing diet for 2 weeks; Sil-4, 4 weeks; Sil-6, 6 weeks; Sil-8, 8 weeks; GPx, glutathione peroxidase; CAT, catalase.

### Serum cytokines levels

**Figure 3:**
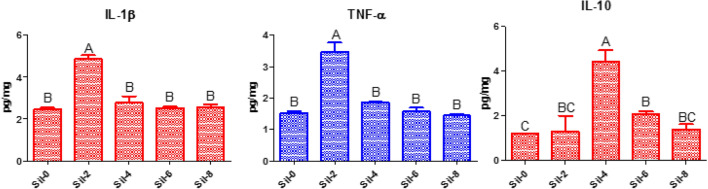
Cytokines levels in the liver tissues of the experimental fish. Different letters indicate significant differences between values at *P* ≤ 0.05. Sil-0, control group; Sil-2, group fed silymarin-containing diet for 2 weeks; Sil-4, 4 weeks; Sil-6, 6 weeks; Sil-8, 8 weeks; IL, interleukin; TNF, tumor necrosis factor.

Cytokines (IL-1β, TNF-α, and IL-10) levels were altered in liver tissues of Nile tilapia fed a silymarin-containing diet. Levels of proinflammatory cytokines were significantly increased at two weeks (IL-1β, 4.87 pg/mg; TNF-α, 3.47 pg/mg) compared to baseline (IL-1β, 2.48 pg/mg; TNF-α, 1.52 pg/mg) and then decreased thereafter. IL-10 levels in hepatic tissues were significantly increased at four weeks (4.43 pg/mg) compared to baseline (1.2 pg/mg) and then decreased at eight weeks (1.4 pg/mg; Fig. 3).

### Infection of experimental fish with *A. hydrophila*

**Figure 4:**
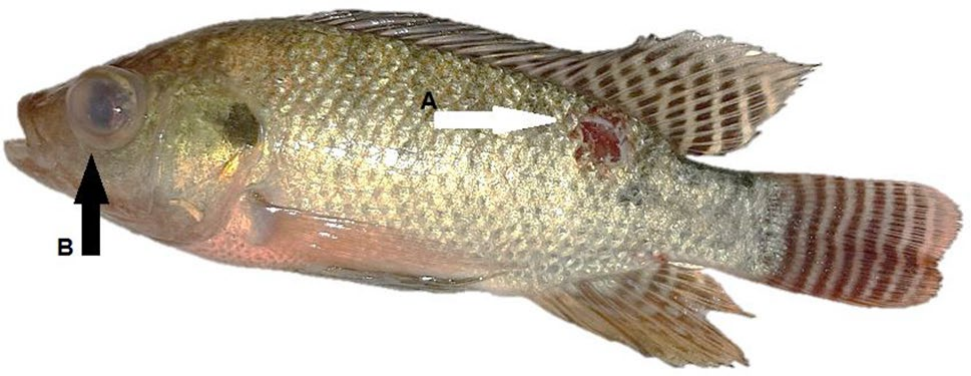
*Oreochromis nioticus* with hemorrhagic ulcers (arrow **A**) and popeye (arrow **B**).

**Figure 5:**
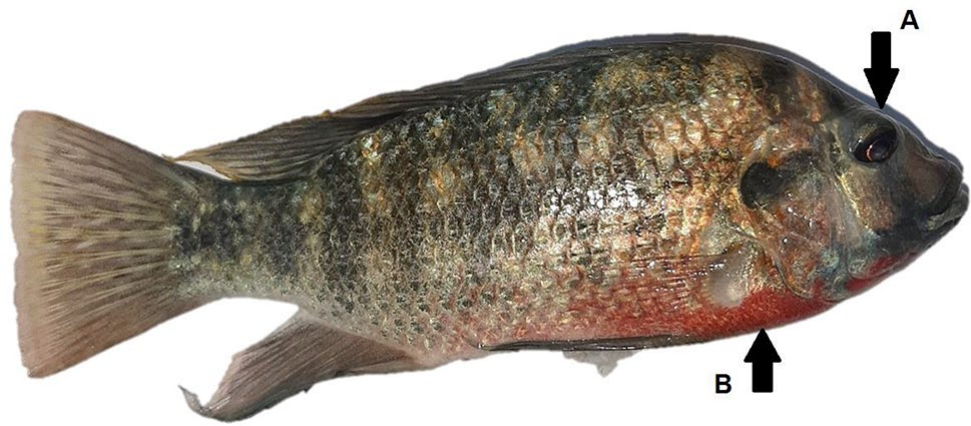
*Oreochromis nioticus* with mild popeye (arrow **A**) and multiple hemorrhagic petechia (arrow **B**).

**Figure 6:**
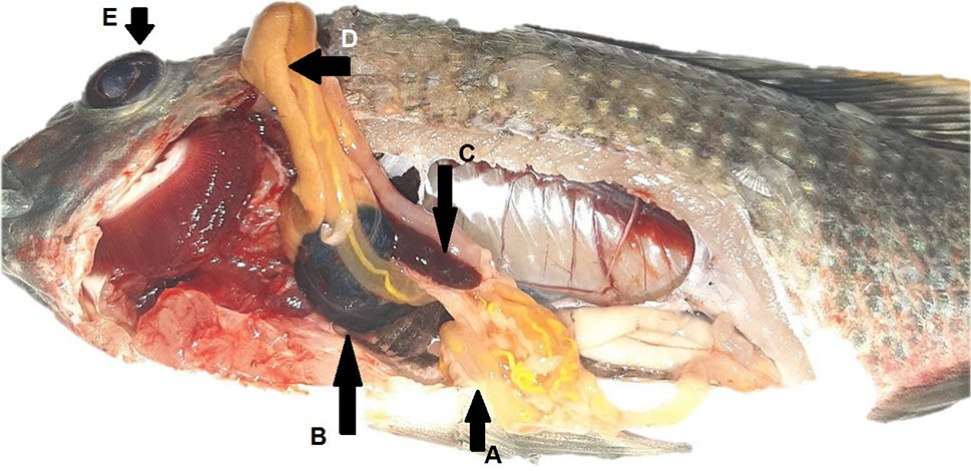
*Oreochromis nioticus* with an empty intestine (arrow **A**), distended gall bladder (arrow **B**), splenomegaly (arrow **C**), yellow-colored liver (arrow **D**), and mild popeye (arrow **E**).

**Figure 7:**
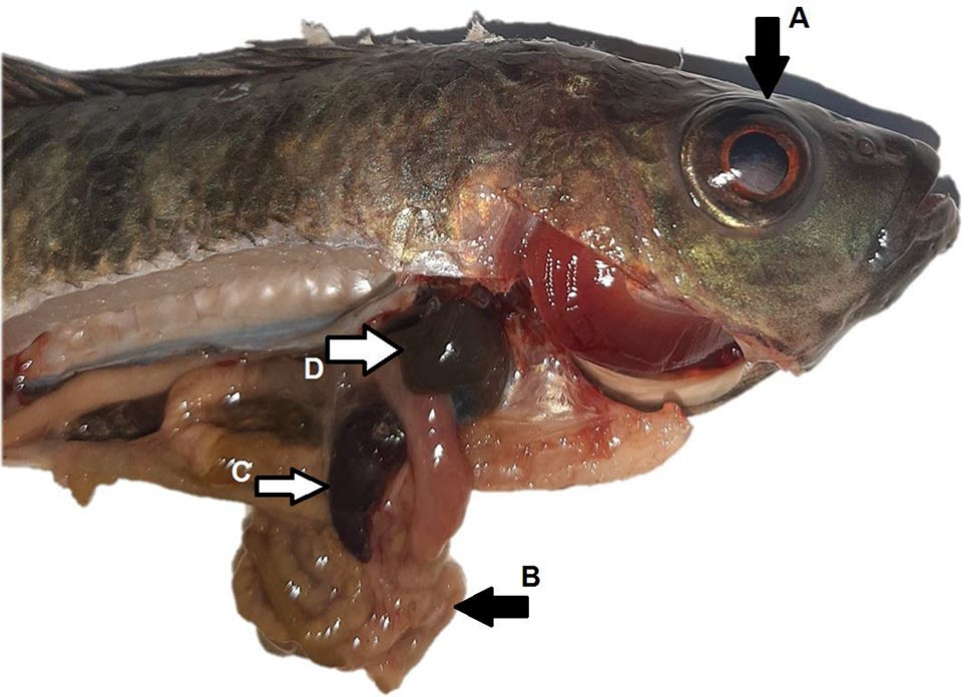
*Oreochromis nioticus* with popeye (arrow **A**), a full intestine (arrow **B**), splenomegaly (arrow **C**), and brown-colored liver (arrow **D**).

Infective signs and post-mortem examinations, Nile tilapia infected with *A. hydrophila* developed multiple hemorrhagic petechia, hemorrhagic ulcers, and pop eye (Figs. 4 and 5). Post-mortem examinations revealed signs of septicemia (Figs. 6 and 7) as splenomegaly and yellow- or brown-colored hepatic tissue. Some fish ad empty intestines (Fig. 6), while others had full intestines (Fig. 7).

Among *O. nioticus* infected with *A. hydrophila*, fish fed a silymarin-containing diet had lower mortality than controls, with silymarin supplementation providing a protection level of 25% at six weeks. Oxytetracycline treatment decreased the MR to 70% compared to 80% in controls. In addition, treatment with oxytetracycline resulted in a relative protection level (RPL) of 37.5% after six weeks of silymarin supplementation as shown in Table 4.

**Table 4:** Mortality rate (MR) and relative protection level (RPL).

Items	Fish no	Silymarin		OTC	
		MR (%)	RPL (%)	MR (%)	RPL (%)
Sil-0	10	80	–	70	12.5
Sil-2	10	80	0	60	25
Sil-4	10	70	12.5	60	25
Sil-6	10	60	25	50	37.5
Sil-8	10	60	25	50	37.5

No., number; MR, mortality rate; RPL, relative protection level; OTC, oxytetracycline.

### Histopathological examination of experimental fish with *A. hydrophila*

**Figure 8:**
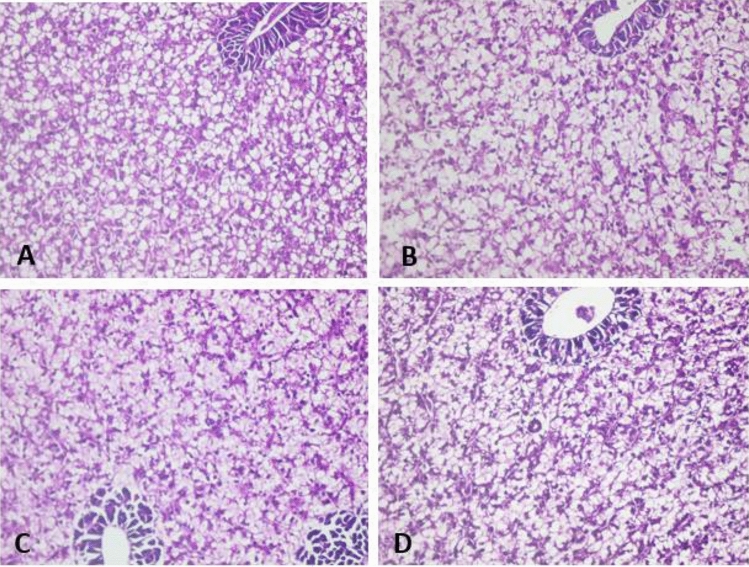
Liver of Sil-2 showed normal Tilapia hepatic tissue criteria (**A**), liver of Sil-2 showed significantly scanty hyaline bodies detection and focal areas of hepatocytes degeneration (**B**). liver of Sil-6 (**C**) and Sil-8 (**D**) neither hyaline bodies nor focal necrotic areas could be detected. Hand E × 400.

After two weeks of silymarin supplementation, there were no changes in the hepatic tissue of Nile tilapia criteria (Fig. 8A), but after four weeks of supplementation, the hepatic tissue revealed scanty hyaline bodies and focal zones of hepatocyte degeneration (Fig. 8B). After six and eight weeks of silymarin supplementation, no hyaline bodies or degenerated areas were observed (Fig. 8C,D).

**Figure 9:**
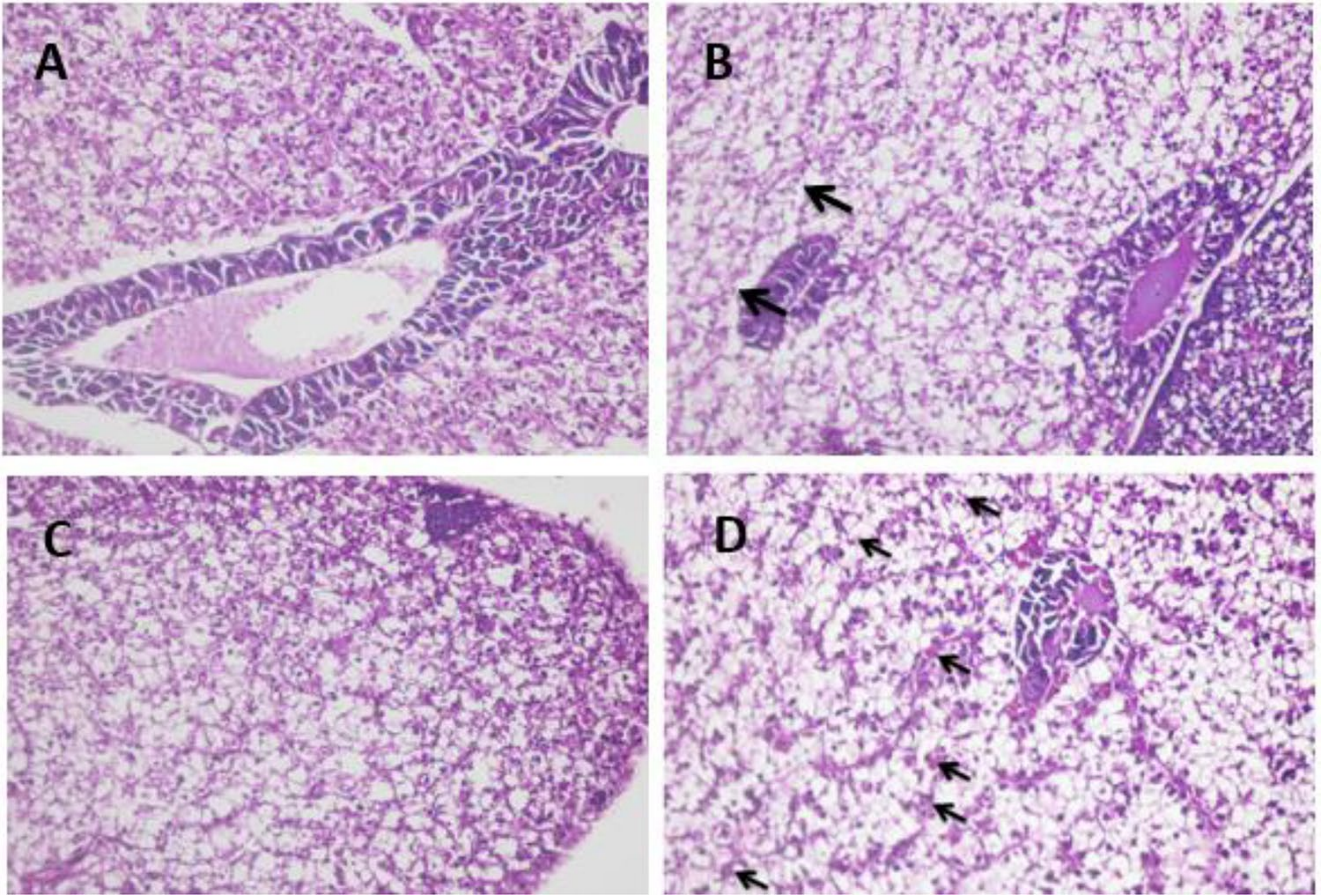
Liver of Sil-2/Challenge showed intra-vascular thrombus formation (**A**) and hemolysis with scant number hyaline bodies (arrows), there is marked vacuolation and necrosis of pancreatic cells (**B**), in addition to foci of necrosis and mono-nuclear cells infiltration (**C**). Liver of Sil-4/Challenge (**D**) showed intra-vascular haemolysis with high incidence of hyaline bodies’ formation. Hand E × 400.

**Figure 10:**
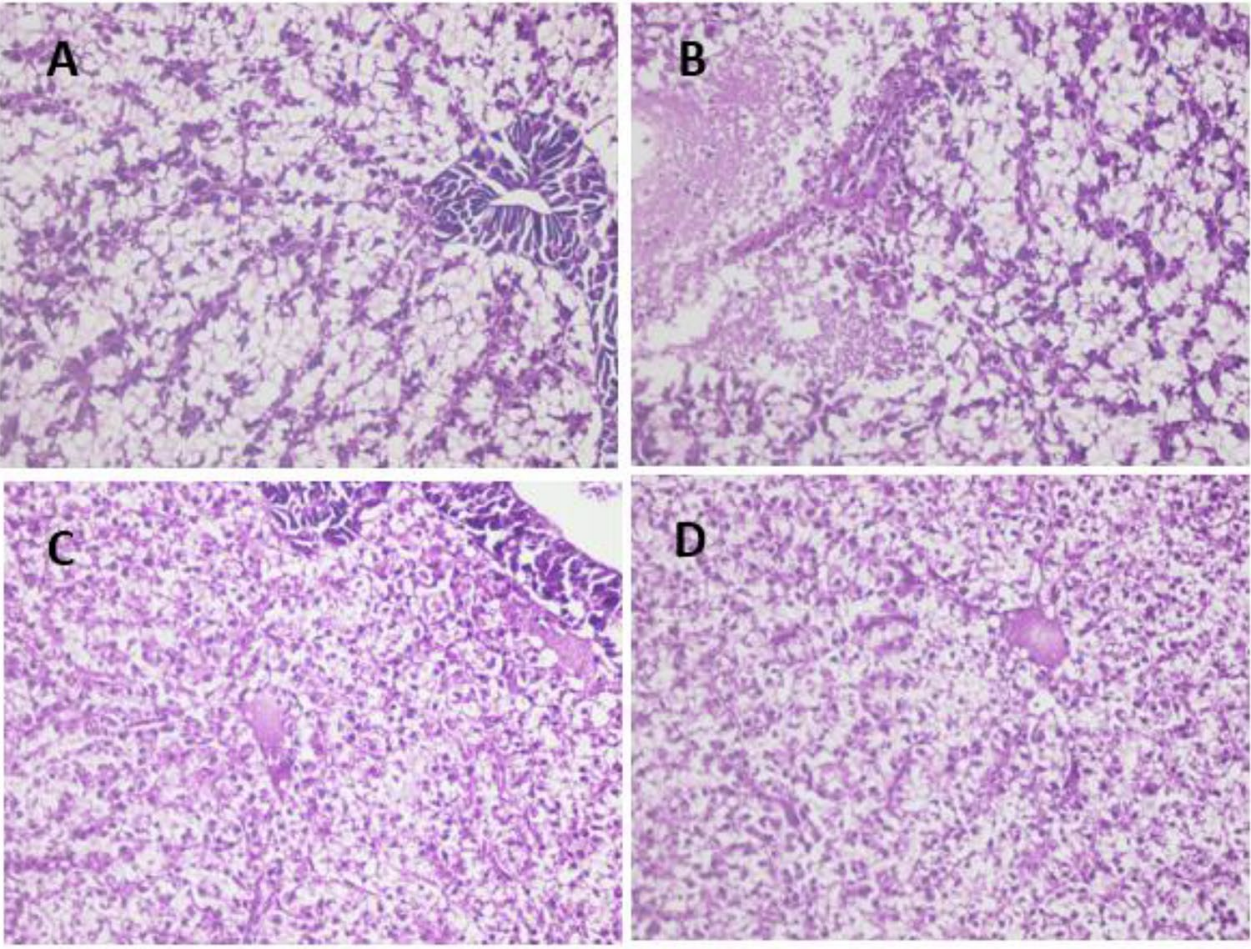
Liver of Sil- 6/Challenge no intra-vascular hemolysis nor thrombus could be detected (**A**), meanwhile multiple areas of hepatocytes lysis was detected (**B**). Liver of Sil-8/Challenge high incidence of lytic areas (**C**, **D**). H and E × 400.

*A. hydrophila* demonstrated septicemic infection, intra-vascular thrombus development, and haemolysis after two weeks of silymarin supplementation followed by challenge (Fig. 9A). Hyaline bodies were shown, and pancreatic cells showed significant vacuolation and even necrosis (Fig. 9B), in addition to necrotic foci (Fig. 9C). At challenge post four weeks of silymarin supplementation, intra- vascular hemolysis could be detected with a high incidence of hyaline bodies’ formation (Fig. 9D). Supplementation with silymarin for six weeks followed by challenge, no intra-vascular hemolysis nor thrombus could be detected (Fig. 10A), meanwhile multiple areas of hepatocytes lysis was detected (Fig. 10B). Challenge after silymarin supplementation for 8 weeks revealed a high incidence of lysis area occurrences, meanwhile, no intra-vascular hemolysis nor thrombus could be detected (Fig. 10C,D). The protection estimation of the silymarin supplementation showed no effect at two and four weeks. At periods of six and eight weeks, the intra-vascular effect of *A. hydrophila* infection (thrombus and hemolysis) was inhibited, meanwhile the cellular degenerative effect could not be improved.

## Discussion

Compared to the control group, Nile tilapia fed a silymarin-containing diet had a higher survival rate after two weeks (95%) until the end of the experimental period (eight weeks). These results demonstrate fish fed a diet supplemented with different doses of silymarin had significantly greater survival compared to fed the control fish^45^.

In the present study, Nile tilapia fed a silymarin-containing diet had improvements in blood indices after four weeks (RBC, 6.45 × 10^3^; WBC, 49.27 × 10^3^; Hb, 14.13 g/dl; PCV, 45.57%) compared to baseline. Values were comparable to the control group at other time points. Similar to our findings, Nile tilapia fed a diet supplemented with silymarin for 45 days have been reported to have higher RBC compared to fish fed a control diet. An increase of WBC count was observed in *Oncorhynchus mykiss* on day 15 of feeding with a dietary silymarin 0.1 g/kg, with higher RBCs on days 15 and 30 in fish fed a dietary silymarin 0.8 or 0.1 g/kg compared to the controls^46^. Conversely, blood indices including WBC, Hb, and PCV were similar between fish fed on dietary silymarin and control diets^23^. These differences between the results of the present study and previous studies may be attributable to the longer duration of feeding with silymarin-supplemented diets as blood indices were comparable to baseline levels after 6 weeks of feeding with a silymarin-containing diet in the present study.

The liver is the primary organ responsible for the metabolism (biotransformation and detoxification) of toxicants including microbial toxins, viruses, heavy metals, aquatic pollutants, pesticides, and chemotherapeutic drugs which may cause organ damage^47,48^. After four weeks of silymarin supplementation, serum ALT levels were significantly decreased compared to fish fed a control diet, while serum AST and ALK were comparable between fish fed a silymarin-containing diet and the control diet throughout the experimental period. Further, serum TG and CH levels were unaffected by silymarin-containing diets; however, serum HDL levels were significantly increased in fish fed a silymarin-containing diet compared to fish fed the control diet. A previous study reported decreased serum AST and ALT levels in Nile tilapia fed a diet with higher *S. marianum* content, with serum AST and ALT levels lowest in Nile tilapia fed a diet supplemented with 10 g/kg fish feed^45^. These decreases in serum AST and ALT levels may be attributable to the strong antioxidant activity of silymarin increasing intracellular levels of glutathione, thereby increasing the elimination of free radicals and inhibition of lipid peroxidation. These effects may protect the cell membranes and ultimately prevent the release of liver enzymes into the circulating plasma. Further, the hepatoprotective properties of silymarin may prevent the transport of xenobiotics across the cell membrane^9,49,50^.

In general, fish with higher serum TP, ALB, and globulin levels are considered to have more robust innate immune responses^51^. After four weeks of silymarin supplementation. Serum proteins were significantly increased compared to baseline values. Further, serum gamma GLO levels were increased in fish fed a silymarin-containing diet compared to fish fed the control diet. Similarly, significant increases in total plasma protein levels have been reported in rainbow trout fed on diets containing silymarin at a dose of 100 and 800 mg/kg compared to the control group^52^. Serum ALB, GLO, and TP levels in Nile tilapia were affected by diets containing *S. marianum* seeds, with TP and GLO content markedly higher in fish fed either diets containing *S. marianum* seeds at 7.5 or 10 g/kg diet compared to other diets. In addition, serum ALB levels were significantly higher in fish fed on diets supplemented with *S. marianum* seeds^45^. On the contrary, no differences in serum TP levels, immunoglobulin levels, agglutinating titers, or antimicrobial activity were observed between Nile tilapia fed a silymarin-supplemented diet and tilapia fed a control diet^23^.

The use of natural herbs or their extracts as feed additives is beneficial for the growth of fish due to their antioxidant, growth-promoting, appetite-stimulating, and immunostimulant properties. This impact is attributed to the presence of bioactive components including flavonoids, phenolics, essential oils, and pigments^7,53,54^. Superoxide dismutase (SOD) and catalase (CAT) are important biomarkers of the antioxidant capacity of cells and are frequently utilized as non-specific immunological indices in fish^55^. In this work, hepatic levels of GPx and CAT, antioxidant enzymes, increased after four weeks of feeding with a dietary silymarin and then returned to baseline levels. A previous study reported the activities of CAT, GST, and SOD were comparable between Nile tilapia fed on a silymarin-containing diet for 6 weeks compared to fish fed a control diet^23^. Silymarin supplementation in the form of *S. marianum* seeds improved antioxidant activity in hepatic tissues and had a direct impact on immune cells^56^. Also, Nile tilapia fed a diet containing *S. marianum* at doses of 7.5 or 10 g/kg had higher SOD activity compared to fish fed other diets, with a trend toward increase of CAT activity in fish provided a diet containing *S. marianum* seeds^45^. These results may be explained by the findings of previous studies^9,47,57^ demonstrating silymarin has cytoprotective properties due to antioxidant and free radical scavenging effects, these authors posited that stress causes the formation of reactive oxygen species (ROS) in organ tissues leading to lipid peroxidation of cell membranes and modification of cell transporters, glycoproteins, estrogenic receptors, and nuclear receptors, as silymarin supplementation is thought to protect against these deleterious impacts of ROS.

Levels of IL-1β, TNF-α, and IL-10 were increased in the hepatic tissues of Nile tilapia fed on dietary silymarin in the present study; however, levels had returned to baseline levels at eight weeks. Dietary silymarin was reported to increase the expression of TNF-α, nitric oxide synthase (iNOS), and interleukins (IL-1β and IL-6) in a dose-dependent manner in rats, further, silymarin at doses up to 10 mg/kg could suppress WBC function, with higher doses between 50 and 250 mg/kg found to stimulate inflammatory processes^10^.

Nile tilapia infected with *A. hydrophila* and fed a silymarin-containing diet had lower mortality compared to the control fish, providing a protection level of 25%. Accordingly, it was reported that 28% of Nile tilapia fed a silymarin-supplemented diet survived streptococcosis while all fish fed a control diet died by 192 h^23^. Similarly, Rutin, a dietary flavonoid, had hepatoprotective effects in silver catfish treated with oxytetracycline, with reduced oxidative stress and apoptosis^58^. Nile tilapia fed on diets containing *Moringa oliefra*, a flavonoid, were protected against oxidative stress in hepatic tissues induced by oxytetracycline treatment^18^.

Oxytetracycline treatment decreased mortality in the present study. In addition, treatment with oxytetracycline after six weeks of feeding with a silymarin supplementation diet provided an RPL of 37.5% compared with 12.5% in fish fed a control diet. Oxytetracycline is an approved therapeutic agent for aquaculture use^59–61^ and oral administration is the most common route of antibiotic administration in aquaculture raising the risk of environmental pollution^62^.

This study has demonstrated that the provision of a silymarin-supplemented diet could reduce the increased antioxidant and immune activities caused by oxytetracycline withdrawal. Accordingly, oral oxytetracycline administration at a dose of at 80 mg/kg biomass/day for 10 consecutive days reduced the antioxidant capacity of liver and muscle tissues and reduced the expression of TNFα and IL-1 in hepatic tissues, indicating that oxytetracycline compromises the innate immunity of *O. niloticus*^63^*.* These authors posited that the cessation of oxytetracycline treatment partially restored the expression of IL-1β. Likewise, low doses of doxycycline have been shown to down-regulate proinflammatory cytokines, including TNF-α and IL-1β, in multiple models^64^. Further, a trend toward a decrease in renal IL-1β expression has been reported in gilthead seabream *(Sparus aurata)* after administration of oxytetracycline at 4 or 8 g/kg feed for 21 days^65^.

In our study, silymarin supplementation could maintain healthy hepatic tissue in Nile tilapia. While some authors detected some drawbacks effect of silymarin supplementation in the form of hepatic vacuolization and necrosis^66,67^ on the other side recorded the improvement of hepatic steatosis by silymarin supplementation. In this study, *A. hydrophila* infection resulted in hepatic degenerative changes, which were previously recorded in the form of necrosis^68^, in association with mononuclear cell infiltrations^69,70^. While some authors^71^ detected the haemosiderin pigment granules in the infected fish tissues, our study recorded the earlier haemolytic changes in the form of intra-vascular blood haemolysis. These changes were attributed to the bacterial toxins and extracellular products such as hemolysin of Aeromonas^72,73^. In the present study, silymarin supplementation could ameliorate the intra-vascular effect of *A. hydrophila* infection however, the cellular degenerative effect could not be improved. For induction of an effective role, silymarin should be administrated in early life stages^74^. In accordance, silymarin with its flavonolignans, flavonoids (taxifolin, quercetin), and polyphenolic molecules^47^ has a hepatoprotective effect mediated by its free radical scavenger effect with modulation of enzymes involved in cellular damage and cirrhosis^74^. Generally, the hepatoprotective effect of silymarin could be summarized; as an anti-inflammatory, antioxidant, anti-proliferative, anti-lipidemic, anti-fibrotic, nuclear expression regulation, mitochondrial membrane stabilization, preserving hepatic mitochondrial bioenergetics, and decreased elevation of AST and ALT in serum^75,76^.

## Conclusion

Silymarin supplementation decreases serum levels of liver enzymes, low-density lipoprotein cholesterol, and cholesterol and significantly increases hepatic levels of glutathione peroxidase and catalase in Nile tilapia. Further, serum TP levels and circulating levels of IL-1β and fish TNF-α were increased, while serum levels of IL-10 were decreased, in tilapia fed a diet containing silymarin. Finally, oxytetracycline treatment provided a RPL of 37.5% against *A. hydrophila* in Nila tilapia fed a silymarin-supplemented diet.

## Data Availability

Data are available on request from the corresponding author.
